# Prospective study of live attenuated vaccines for patients receiving immunosuppressive agents

**DOI:** 10.1371/journal.pone.0240217

**Published:** 2020-10-01

**Authors:** Koichi Kamei, Isao Miyairi, Kenji Ishikura, Masao Ogura, Kensuke Shoji, Katsuhiro Arai, Reiko Ito, Toshinao Kawai, Shuichi Ito

**Affiliations:** 1 Division of Nephrology and Rheumatology, National Center for Child Health and Development, Tokyo, Japan; 2 Division of Infectious Diseases, National Center for Child Health and Development, Tokyo, Japan; 3 Department of Pediatrics, Kitasato University School of Medicine, Kanagawa, Japan; 4 Division of Gastroenterology, National Center for Child Health and Development, Tokyo, Japan; 5 Department of General Pediatrics, National Center for Child Health and Development, Tokyo, Japan; 6 Division of Immunology, National Center for Child Health and Development, Tokyo, Japan; 7 Department of Pediatrics, Yokohama City University, Kanagawa, Japan; Universidad de Navarra, SPAIN

## Abstract

Patients receiving immunosuppressive agents are at risk of life-threatening infections. However, live vaccines are generally contraindicated in them. We conducted a prospective study regarding live attenuated vaccines for them. Patients elder than one year of age with immunosuppressive agents who showed negative or borderline antibody titers (virus-specific IgG levels < 4.0) against one or more of measles, rubella, varicella, and mumps and fulfilled the criteria (CD4 cell counts ≥ 500/mm^3^, stimulation index of lymphocyte blast transformation by PHA ≥ 101.6, serum IgG level ≥ 300 mg/dl, no steroid use or prednisolone < 1 mg/kg/day or < 2 mg/kg/2 days, trough levels of tacrolimus or cyclosporine were < 10 ng/ml or < 100 ng/ml and under good control of primary disease) were enrolled. Sixty-four vaccinations were administered to 32 patients. The seroconversion rates for measles, rubella, varicella, and mumps were 80.0%, 100.0%, 59.1%, and 69.2%, respectively. No life-threatening adverse events were observed, although one patient suffered from vaccine-strain varicella who showed cellular and humoral immunodeficiency (CD4 cell counts = 511/mm^3^, stimulation index of lymphocyte blast transformation by PHA = 91.1, serum IgG level = 208 mg/dl). This girl was immunized before we established the criteria for vaccination. Immunization with live attenuated vaccines for patients receiving immunosuppressive agents might be effective and safe if their cellular and humoral immunological parameters are within normal levels. However, determining the criteria for vaccination by immunological parameters should be established to guarantee the safety of live vaccines in the future.

Clinical Trial Registration: UMIN Clinical Trials Registry (UMIN-CTR) UMIN000007710. The date of registration: 2012/4/13.

## Introduction

Viral infections, such as varicella and measles, sometimes cause fatal complications in children who are treated with immunosuppressive agents. Sometimes, recurrent of disease might observed in them, such as the relapse of nephrotic syndrome, after viral infections. Therefore, protection from infection by vaccination is necessary for these vaccine-preventable diseases. However, administration of live vaccine is generally contraindicated in patients receiving immunosuppressive agents. To date, several case series examining immunization using live attenuated vaccines for patients receiving immunosuppressive agents, mainly solid organ transplant recipients, have been published [1–14]. In these reports, live attenuated vaccines were generally effective and safe, and no life-threatening adverse events were observed.

We previously published a prospective study concerning live attenuated vaccines administered to patients with nephrotic syndrome receiving immunosuppressive agents (116 vaccinations in 60 patients) from May 2011 to January 2017 in our center [15]. The present study was conducted following the same protocol in patients with medical conditions other than nephrotic syndrome, such as inflammatory bowel disease, rheumatic disease, hepatic disease, and liver or kidney transplants, to investigate vaccine efficacy and safety in these patients.

## Materials and methods

### Study design and patient population

This prospective study was performed from May 2012 to April 2018 at the National Center for Child Health and Development (NCCHD) in Tokyo, Japan. It was performed in accordance with the Declaration of Helsinki and was approved by the ethics committee of the National Center for Child Health and Development (No. 452). This study was also registered under the University Hospital Medical Information Network–Clinical Trials Registry (Registration date, April 13th, 2012; registration number, UMIN 000007710; clinical trial number, R000009090).

Inclusion criteria for eligible patients are listed in Table 1. We established the criteria for phytohemagglutinin (PHA) stimulation index and serum IgG levels in July 2013 after we encountered a four-year-old girl with a renal transplant who contracted vaccine-strain varicella (details are shown in the Results section). Virus-specific antibody titers were measured using a commercially available enzyme immunoassay (EIA) for measles, rubella, varicella, and mumps. Negative (–), borderline (±), and positive (+) antibody titers were defined based on EIA values of IgG levels < 2.0, 2.0–3.9, and ≥ 4.0, respectively.

**Table 1:** Inclusion criteria for eligible patients.

1. Age ≥ 1 year old.
2. Negative or borderline antibody titers against measles, rubella, varicella or mumps.
3. Under treatment with one or two immunosuppressive agents (CsA, Tac, MMF, MZR, AZP, EVR or MTX).
4. Normal cellular immunity
CD4 cell counts ≥ 500/mm^3^
Normal lymphocyte blast transformation by PHA (stimulation index ≥ 101.6)
5. Serum IgG level ≥ 300 mg/dl
6. No steroid use or prednisolone < 1 mg/kg/day or < 2 mg/kg/2 days
7. Trough levels of tacrolimus < 10 ng/ml
Trough levels of cyclosporine < 100 ng/ml
8. Under good control of primary disease.
9. Difficult to discontinue immunosuppressants because of primary disease.
10. Written informed consent was obtained from patients or families.

CsA, cyclosporine; Tac, tacrolimus; MMF, mycophenolate mofetil; MZR, mizoribine; AZP, azathioprine; EVR, everolimus; MTX, methotrexate; PHA, phytohemagglutinin.

### Study protocol

After written informed consent was obtained from patients or their guardians, the immunological parameters (CD4 cell count, PHA stimulation index, and serum IgG level) were assessed. We have an intramural committee for live attenuated vaccines for patients receiving immunosuppressive agents, which consists of physicians in the Divisions of Infectious Diseases, Immunology, Nephrology and Rheumatology, and Gastroenterology, and the Department of General Pediatrics in our center for monitoring the efficacy and safety of vaccinations. This prospective study was conducted under the supervision of this committee. If the parameters met the inclusion criteria, the intramural committee discussed the indications for vaccination.

After the committee’s approval was obtained, vaccination was administered. We used a freeze-dried live attenuated measles and rubella (MR) combined vaccine (Schwartz FF strain for measles and the TO-336 strain for rubella, Takeda, Osaka, Japan), a freeze-dried live attenuated varicella vaccine (Oka strain, Biken, Osaka, Japan), and a freeze-dried live attenuated mumps vaccine (Torii strain, Takeda, Osaka, Japan until October 2012 and Hoshino strain, Kitasato Daiichi Sankyo, Saitama, Japan from November 2012). Each vaccination was specified as single dose in this study protocol. We examined the antibody titers two months after vaccination. A positive antibody titer (virus-specific IgG levels ≥ 4.0 EIA units) was defined as seroconversion (seropositivity, responder) and a borderline or negative antibody titer (virus-specific IgG levels < 4.0 EIA units) was defined as non-responder. Adverse events after vaccination were monitored by clinic visits or phone calls and were discussed by the committee. Patients who failed to respond to the first vaccine dose in this study (non-responders) or who became seronegative after a positive response (secondary vaccine failure) were offered additional doses of vaccine.

### Study endpoints

The primary endpoint of this study was the seroconversion rate at two months after the initial vaccination in this study. The secondary endpoints included adverse events and breakthrough infections. Breakthrough infection is defined as infection of wild-type strain after vaccination. All these data were analyzed separately for each virus.

### Ethical approval

This study was performed in accordance with the Declaration of Helsinki and was approved by the ethics committee of the National Center for Child Health and Development (No. 452). Written Informed consent was obtained from all participants or their families before the study enrollment.

## Results

### Patient characteristics

**Fig 1:**
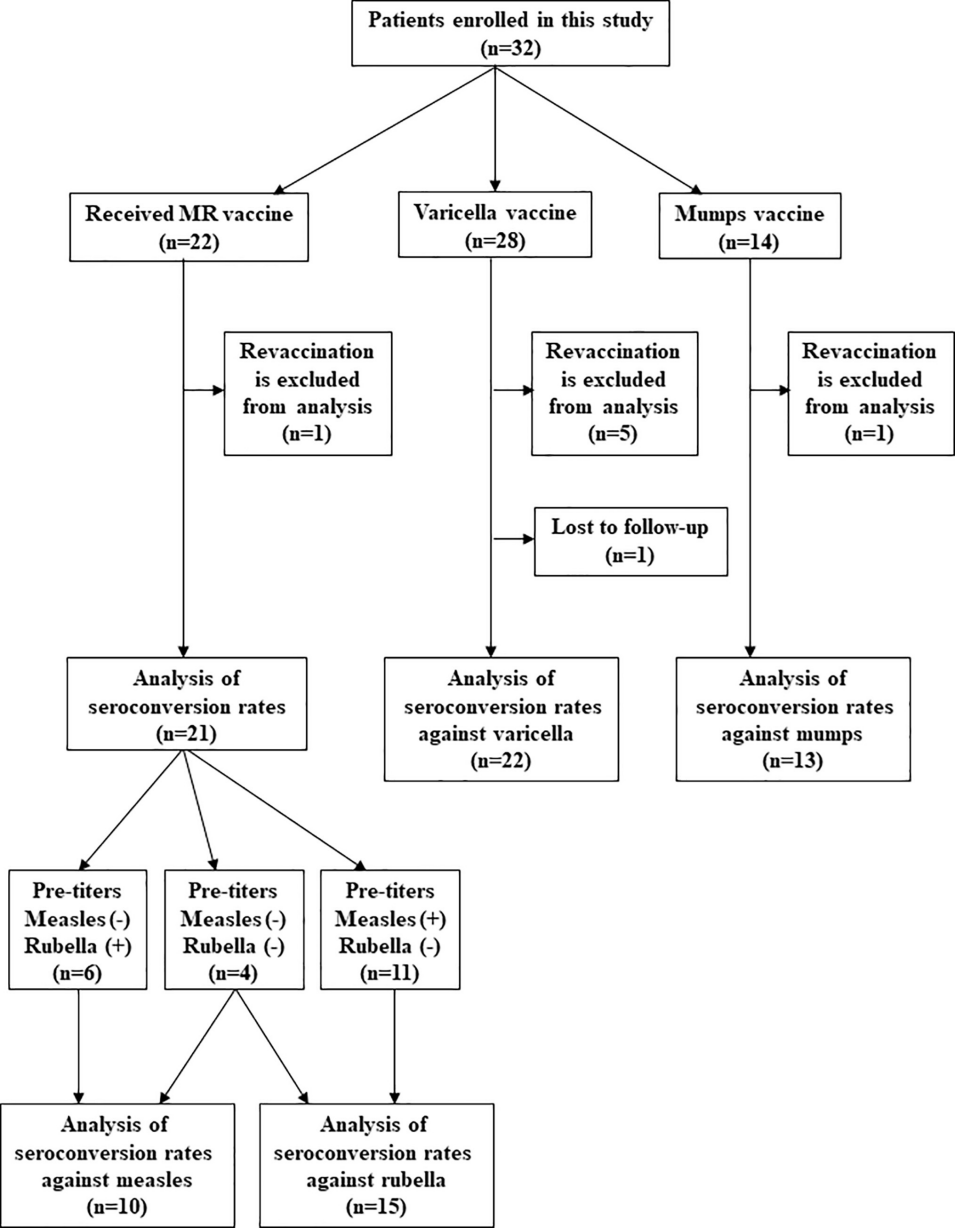
Flow diagram of patient population.

Sixty-four vaccinations were administered to 32 patients (Table 2). Fifteen patients received once vaccine and 17 patients received more than two vaccines. Inflammatory bowel disease was the most primary disease and Rheumatic disease was the second. The varicella vaccine was the most frequently administered vaccine. The number of patients with steroid use at vaccination was 33 (51.6%). Flow diagram of this study cohort is shown in Fig 1.

**Table 2:** Patient characteristics.

Patients	32
Male sex	15 (46.9%)
Primary disease	
Inflammatory bowel disease	12 (37.5%)
Rheumatic disease	10 (31.3%)
Kidney transplant	4 (12.5%)
Liver transplant	3 (9.4%)
Hepatic disease	2 (6.3%)
IgA nephropathy	1 (3.1%)
Number of vaccinations	64
MR vaccine	22 (34.4%)
Varicella vaccine	28 (43.8%)
Mumps vaccine	14 (21.9%)
Number of initial vaccinations	57
MR vaccine	21 (36.8%)
Varicella vaccine	23 (40.4%)
Mumps vaccine	13 (22.8%)
Age at vaccination (y)	5 (1–25)
CD4 cell count at vaccination (/mm^3^)	1104.9±414.7
PHA stimulation index at vaccination	321.4±165.2
Serum IgG at vaccination (mg/dL)	932.8±409.6
Immunosuppressive agents at vaccination	
Calcineurin inhibitors (CsA, Tac)	7 (10.9%)
Antimetabolite agents (MMF, MZR, AZP, EVR, MTX)	41 (64.1%)
Calcineurin inhibitors + antimetabolite agents	13 (20.3%)
Two antimetabolite agents	3 (4.7%)
Steroid use at vaccination	33 (51.6%)

Data were shown as number (%), mean ± standard deviation or median (range).

MR, measles and rubella; PHA, phytohemagglutinin; CsA, cyclosporine; Tac, tacrolimus; MMF, mycophenolate mofetil; MZR, mizoribine; AZP, azathioprine; EVR, everolimus; MTX, methotrexate.

The primary analysis (seroconversion rates against each virus) were conducted in the initial vaccination in this study. Adverse events were monitored in all vaccinations.

### Seroconversion rates after the initial vaccination in this study

Seroconversion rates after vaccination for the initial vaccination in this study was shown in Table 3. The numbers of patients for analysis of seroconversion are different from the number of vaccinations in Table 2. One reason is that MR vaccine was indicated for patients with negative or borderline antibody titers in either measles or rubella before vaccination and Table 3 includes only viruses which showed negative or borderline antibody titers. Another reason is that Table 3 includes only the initial vaccinations in this study, although Table 2 includes revaccinations (Fig 1).

**Table 3:** Seroconversion rates after initial vaccination in this study.

	Measles	Rubella	Varicella	Mumps
Number of vaccinations	10	15	22	13
Seroconversion	8 (80.0%)	15 (100.0%)	13 (59.1%)	9 (69.2%)
Vaccine failure				
Borderline (±)	1 (10.0%)	0 (0.0%)	5 (22.7%)	2 (15.4%)
Negative (±)	1 (10.0%)	0 (0.0%)	3 (13.6%)	2 (15.4%)
Antibody titer after vaccination				
Mean ± SD	34.3 ± 39.1	55.0 ± 67.7	7.0 ± 6.8	7.1 ± 7.1
Median (range)	21.4 (<2.0–135.0)	32.5 (4.5–275.0)	5.2 (<2.0–27.6)	4.7 (<2.0–27.2)

Data were shown as number (%), mean ± standard deviation or median (range).

The seroconversion rates for measles, rubella, varicella, and mumps were 80.0%, 100.0%, 59.1%, and 69.2%, respectively. For the mumps vaccine, the seroconversion rates for the Torii and Hoshino strains were the same

### Adverse events

A four-year-old girl with a renal transplant suffered from varicella 42 days after varicella vaccination. She was under treatment with tacrolimus, mycophenolate mofetil, and methylprednisolone. Her immunological parameters were the following: CD4-positive cells, 511/mm^3^; PHA stimulation index, 91.1 (normal value, ≥ 101.6); serum IgG level, 208 mg/dL. She was admitted to our center, received acyclovir, and recovered promptly. Later, the analysis of vesicular content revealed vaccine-strain varicella (Oka vaccine strain) by allelic discrimination real-time PCR [16].

Other adverse events were non-specific and were not directly linked to vaccination (Table 4). The rash observed in three patients after varicella vaccinations did not resemble varicella. Real-time polymerase chain reaction of saliva in the patient that developed parotitis after mumps vaccination was negative for mumps virus [17].

**Table 4:** Adverse events.

Events	MR vaccine	Varicella vaccine	Mumps vaccine
	(22 vaccination)	(28 vaccination)	(14 vaccination)
Fever	3		
Cough and nasal discharge	1		
Cellulitis	1		
Rash		2	
Fever and rash		1	
Diarrhea		1	
Transient liver dysfunction		1	
Vaccine strain infection		1^a)^	
Parotitis			1^b)^

a) Varicella of vaccine strain.

b) Real-time polymerase chain reaction of saliva was negative for mumps virus.

### Breakthrough infections after vaccination during the study period

A five-year-old boy with Crohn disease under treatment with azathioprine contracted breakthrough varicella of the wild-type strain six months after varicella vaccination. He recovered promptly with oral acyclovir treatment.

## Discussion

In this prospective study, we evaluated the efficacy and safety of live attenuated vaccines for patients receiving immunosuppressive agents. The seroconversion rates were 80.0% for measles, 100.0% for rubella, 59.1% for varicella, and 69.2% for mumps, which showed similar results to the former study of patients with nephrotic syndrome [15]. More patients in this present study were using steroid (51.5%) than those in the former study (3.4%), although immunological parameters were similar. No life-threatening adverse events were observed. Immunization with live attenuated vaccines may be effective and safe in patients under treatment with immunosuppressive agents who have cellular and humoral immunologic parameters within clinically acceptable ranges regardless of type of disease.

One patient contracted vaccine-strain varicella. This patient showed a PHA stimulation index of 91.1 (normal value, ≥ 101.6) and a serum IgG level of 208 mg/dL, which suggested cellular and humoral immunodeficiency. Before we encountered this case, we did not have appropriate criteria concerning PHA stimulation index and serum IgG levels. However, after this vaccine-strain varicella case, we established the criteria as PHA stimulation index ≥ 101.6 and serum IgG level ≥ 300 mg/dL. No viral infections by vaccine strain were observed since then. In cases of severe cellular immunodeficiency, live vaccines are contraindicated due to the risk of fatal viral infections by vaccine strain [18–20]. In the review of 675 vaccinations in 317 patients in 15 case series or case reports [1–15], viral infections by vaccine strain were observed in 20 cases (3.0%), consisting of 18 patients in 287 varicella vaccines (6.3%) and 2 patients in 121 mumps vaccines (1.7%). The probability of viral infection by vaccine strain might be higher in patients under treatment with immunosuppressive agents than that in healthy children. There is a possibility that patients treated with immunosuppressive agents might show cellular immunodeficiency in some patients. However, vaccine-strain varicella can be treated by antiviral drugs, such as acyclovir and valaciclovir. We believe that determining the criteria for vaccination by immunological parameters should be established to guarantee the safety of live vaccines in the future.

We could show the efficacy and safety of attenuated live vaccines in patients with nephrotic syndrome under treatment with immunosuppressive agents in the former study (116 vaccinations in 60 patients) [15] and in similarly treated patients with other diseases, such as inflammatory bowel disease and rheumatic disease, in this study (64 vaccinations in 32 patients). In a total of 180 vaccinations in 92 patients in our two studies, viral infection by vaccine strain was observed in only one patient (0.5%) and no life-threatening adverse events were observed.

The present recommendation for the prevention of infection, such as measles and varicella, in immunocompromised individuals is administration of antiviral drugs or intravenous immunoglobulin infusion. Despite the data of our two prospective studies, the safety of live vaccines for patients receiving immunosuppressive agents still remains controversial and we have to collect more information about efficacy and safety. We are currently conducting a nationwide survey of live attenuated vaccines for patients treated with immunosuppressive agents in Japan. According to the results of this nationwide study, we would like to consider the revision of the criteria for “contraindication” in the package inserts for the use of live attenuated vaccines in patients under treatment with immunosuppressive agents in the near future.

## Supporting information

S1 File Study protocol (English). (DOCX)

S2 File Study protocol (Japanese). (DOCX)

S3 File TREND checklist. (PDF)
