# Supplementary material for: Prospective study of live attenuated vaccines for patients receiving immunosuppressive agents
Source: PLoS One. 2020 Oct 1;15(10):e0240217. doi: 10.1371/journal.pone.0240217 (PMC7529194; doi:10.1371/journal.pone.0240217)
Supplement: S1 File — (DOCX) [file pone.0240217.s001.docx]

**Prospective study of live attenuated vaccines for patients with immunosuppressive agents**

**1. Project summary**

Vaccination with live attenuated vaccines is contraindicated during immunosuppressant treatment. However, patients taking immunosuppressants are at high risk of developing severe forms of viral infections such as varicella and measles, and they should undergo immunization with live vaccines to acquire immunity, if possible. This is a single-center, prospective, interventional study. Patients who are either (–) or (±) for antibodies to any of measles, rubella, varicella, or mumps (enzyme immunoassay IgG <4) are included. After immune analysis (CD4 cell counts, phytohaemagglutinin lymphocyte stimulation testing, and serum immunoglobulin G) is conducted, they are immunized with the live attenuated vaccine (measles-rubella vaccine, varicella vaccine, or mumps vaccine). Vius antibody titer for the vaccine concerned and adverse events will be assessed 2–6 months later.

**2. Principal Investigator**

Koichi Kamei, MD, PhD

Division of Nephrology and Rheumatology, National Center for Child Health and Development

2-10-1 Okura, Setagaya-ku, Tokyo 157-8535, Japan

Tel.: +81-3-5494-7128; Fax: +81-3-5494-7909; E-mail: [kamei-k@ncchd.go.jp](mailto:kamei-k@ncchd.go.jp)

**3. Co-investigators**

Masao Ogura^1^, Isao Miyairi^2^, Kensuke Shoji^2^, Katsuhiro Arai^3^, Reiko Ito^4^, Toshinao Kawai^5^, Kenji Ishikura^6^, Shuichi Ito^7^

1) Division of Nephrology and Rheumatology, National Center for Child Health and Development, Tokyo, Japan

2) Division of Infectious Diseases, National Center for Child Health and Development, Tokyo, Japan

3) Division of Gastroenterology, National Center for Child Health and Development, Tokyo, Japan

4) Department of General Pediatrics, National Center for Child Health and Development, Tokyo, Japan

5) Division of Immunology, National Center for Child Health and Development, Tokyo, Japan

6) Department of Pediatrics, Kitasato University School of Medicine, Kanagawa, Japan

7) Department of Pediatrics, Yokohama City University, Kanagawa, Japan

**4. Study Background**

The Red Book of the American Academy of Pediatrics^1)^ states that vaccination with live attenuated vaccines should be avoided until 3 months after the withdrawal of immunosuppressants. The package inserts for the immunosuppressants approved for use in Japan (tacrolimus, cyclosporine, mizoribine, azathioprine, mycophenolate mofetil, and methotrexate) also list live attenuated vaccines as drugs prohibited for concomitant use.

Patients undergoing immunosuppressive therapy and immunocompromised patients are at high risk of developing serious forms of viral infections. Varicella in particular may cause multiple organ failure due to organ damage in immunosuppressed patients. In Japan, unlike the United States, outbreaks of measles and varicella are not uncommon, and these viral infections pose a constant risk. It is our task to protect children who must take immunosuppressants long-term against such viral infections.

Vaccination with live attenuated vaccines of patients undergoing immunosuppressant therapy has already been widely studied^2)-11)^. All of the patients were taking immunosuppressants, in most cases after organ transplantation, and 146 patients acquired antibodies from a total of 192 vaccinations, an antibody acquisition rate of 76%. Adverse events from those 192 vaccinations included 1 case of acute rejection (0.5%), 8 of vaccine-induced viral infection (7 cases of varicella and 1 of mumps) (4.2%), 5 of pyrexia (2.6%), and 8 of local reaction (4.2%), with no fatal adverse events. In general, cases of vaccine-induced viral infections were milder than normal cases.

Intractable nephrotic syndrome (steroid-resistant nephrotic syndrome or frequently relapsing nephrotic syndrome) accounts for approximately 40% of all cases of nephrotic syndrome and tends to occur at a comparatively younger age, with many patients unvaccinated by live vaccines such as the freeze-dried live attenuated combination measles and rubella vaccine or the freeze-dried live attenuated varicella vaccine. Intractable nephrotic syndrome often relapses repeatedly when immunosuppressants such as cyclosporine and mizoribine are tapered or withdrawn, making immunosuppressant withdrawal extremely difficult. Kidney transplant patients all undergo vaccination with live vaccines before undergoing transplantation, which is not performed until elevated antibody levels have been confirmed. Post-transplantation, they must continue taking immunosuppressants for the rest of their lives. Continued immunosuppressive therapy often results in the subsequent loss of antibodies, exposing them to the risk of viral infection. To protect such children who cannot undergo immunosuppressant withdrawal against viral infection, it is essential that they undergo vaccination with live attenuated vaccines while their condition is stable.

**5. Study Objective**

The objective of this study is to use live attenuated vaccines for patients using immunosuppressants for conditions such as kidney disease, rheumatic disease, gastroenterological disease, and kidney transplantation, who have maintained a certain level of immune function and whose condition is stable, and to evaluate their efficacy and safety.

**6. Study Design**

This is a single-center, prospective, interventional study.

**7. Subjects**

Target Diseases

Kidney disease (nephrotic syndrome, chronic glomerulonephritis, etc.)

Kidney transplantation

Rheumatic disease (systemic lupus erythematosus, juvenile rheumatoid arthritis, etc.)

Gastroenterological disease (inflammatory bowel disease, etc.)

Liver disease (autoimmune hepatitis, etc.)

Other diseases

Vaccination Conditions

1. At least 1 year old
2. Taking one or more of the following immunosuppressants: tacrolimus, cyclosporine, mizoribine, azathioprine, mycophenolate mofetil, methotrexate, or everolimus
3. Cellular immunity markers within normal limits
   - - - CD4 cell count ≥500/mm^3^
       - Phytohaemagglutinin (PHA) lymphocyte blastogenic response stimulation index ≥101.6
4. Serum immunoglobulin G (IgG) ≥300 mg/dL
5. Steroid dose below prednisolone 1 mg/kg/daily or 2 mg/kg/alternate days
6. Tacrolimus trough value <10 ng/mL or cyclosporine trough value <100 ng/mL
7. For kidney transplant recipients, at least 1 year elapsed since the transplant, and no acute rejection response within the past 6 months
8. The underlying condition is stable, and there is considered to be little risk of its exacerbation by vaccination
9. Immunosuppressant withdrawal is problematic
10. The Committee to Evaluate Vaccination of Patients Taking Immunosuppressants with Live Attenuated Vaccines determined that vaccination is indicated

**8. Methods**

With the consent of the patient or the patient’s family, antibody titers to four viruses (measles, rubella, varicella, and mumps) and the immune analysis is conducted. A full explanation will be provided of the advantages (antibody acquisition) and disadvantages (the possibility of developing vaccine-induced viral infection and serious side effects) of vaccination for viruses for which patients have yet to acquire antibodies (– or ±, or IgG <4.0 on enzyme-linked immunosorbent assay [ELISA]), as well as the fact that it is listed as contraindicated on the drug package insert. After confirming that this content has been fully understood, informed consent will be obtained in writing. After approval by the Committee to Evaluate Vaccination of Patients Taking Immunosuppressants with Live Attenuated Vaccines, vaccination will be carried out with one or more of the following live attenuated vaccines: freeze-dried live attenuated combination measles and rubella vaccine, freeze-dried live attenuated measles vaccine, freeze-dried live attenuated varicella vaccine, or freeze-dried live attenuated mumps vaccine. Because measles and varicella have a particularly high fatality risk during immunosuppressive therapy, these vaccinations will be proactively administered. Antibody acquisition will be assessed at least 2–6 months after vaccination. IgG ≥4.0 on ELISA (+ or greater) will be considered as indicating antibody acquisition and assessed as effective.

Adverse event evaluation will include the assessment of immediate allergic reaction within 24 h and the occurrence of vaccine-induced viral infection. Post-vaccination safety will be assessed by the Committee to Evaluate Vaccination of Patients Taking Immunosuppressants with Live Attenuated Vaccines for all patients. If a serious adverse event occur, this committee will immediately convene and respond appropriately, having immediately submitted a report to the ethics committee. It will also consider the necessity of discontinuing the study as a whole.

Immunological Assessment

1. Humoral immunity
   - - - Provide pre-vaccination serum IgG level (vaccination permissible at 300 mg/dL)
       - Antibody titers for measles, rubella, varicella, and mumps (IgG levels on ELISA): provide values pre-vaccination and 2–6 months after vaccination
2. Cellular immunity
   - - - Provide pre-vaccination CD4-positive cell count (vaccination permissible at ≥500/mm^3^)
       - Provide results of pre-vaccination PHA lymphocyte stimulation test (vaccination permissible at stimulation index ≥101.6)

**9. Endpoints**

1. Primary endpoints
2. Antibody acquisition rates for each type of vaccine
3. Adverse events for each type of vaccine
4. Secondary endpoints
5. Analysis of the cause in cases of vaccine failure
6. Analysis of the cause in cases of adverse events
7. Effectiveness in preventing viral infections

**10. Anticipated Outcomes**

If its efficacy and safety are demonstrated in this study, vaccination with live vaccines of patients taking immunosuppressants may become feasible, enabling children to be protected against the threat of viral infection.

**11. Study Period**

May 1, 2012 to April 30, 2018

**12. Ethics**

1. Voluntary nature of study participation and right of withdrawal

Study participation is decided voluntarily by the patient. The doctor responsible for the patients’ treatment will explain the nature of the study to them and obtain their consent. The information sheet and consent form will be used in this process. Patients who wish to withdraw may do so at any time. If this is before their data have been collated, these data may not be used. Patients who do not participate in the study will not be placed at any disadvantage during treatment.

1. Informed consent concerning the advantages and disadvantages of study participation

When explaining the study, the doctor responsible for the patient's treatment will explain the following advantages and disadvantages. Vaccination will only be performed after each individual patient has been fully scrutinized, it has been judged that in this particular case the advantages outweigh the disadvantages, and that each patient’s family has fully understood the disadvantages.

Advantages

By undergoing vaccination, the patient can acquire antibodies to viruses and have a decreased risk of infection.

Disadvantages

- 1. There is a possibility that vaccine-induced viral infection may occur.
  2. There is a possibility of extremely serious side effects.
  3. Depending on the disease, the underlying condition may be aggravated (e.g. nephrotic syndrome relapse, rejection of transplanted kidney).
  4. The antibody acquisition rate may be low because the patient is taking immunosuppressants.

**13. Costs and Liability for Indemnity/Compensation**

Study participants are responsible for paying the costs of vaccination. The costs of the pre-vaccination immune tests and tests to ascertain the effectiveness of vaccination are covered by regular health insurance. Should any health injury occur, the hospital will provide the necessary care and treatment in an appropriate manner. As compensation for serious adverse events, for the duration of the study, patients will be enrolled in Sompo Japan's clinical study indemnity liability insurance and the insurance premium will be paid out of a grant from the National Center for Child Health and Development (grant number 24-10).

**14. Protection of Personal Information and Handling of Study Results**

Due care will be taken to protect privacy, and when the study outcomes are published, no information whatsoever will be included that might identify patients, with anonymization used to protect personal information. Patient samples will also be anonymized by the creation of a patient ID correspondence table, and this correspondence table will be managed by the hospital's personal information manager in such a way that it is not accessible by other people. When the study outcomes are reported, only anonymized results will be used, and personal information will not be divulged.

**15. Reference**

1. Red Book. 2009 report of the Committee on Infectious Disease. American Academy of Pediatrics.
2. Danerseau AM, Robinson JL. Efficacy and safety of measles, mumps, rubella and varicella live viral vaccines in transplant recipients receiving immunosuppressive drugs. World J Pediatr 2008 ; 4 : 254-258
3. Rand EB, McCarthy CA, Whitington PF. Measles vaccination after orthotopic liver transplantation. J Pediatr 1993 ; 123 : 87-89
4. Zamora I, Simon JM, Da Silva ME, Piqueras AI. Attenuated varicella virus vaccine in children with renal transplants. Pediatr Nephrol 1994 ; 8 : 190-192
5. Kano H, Mizuta K, Sakakihara Y, Kato H, Miki Y, Shibuya N, Saito M, Narita M, Kawarasaki H, Igarashi T, Hashizume K, Iwata T. Efficacy and safety of immunization for pre- and post- liver transplant children. Transplantation 2002 ; 74 : 543-550
6. Levitsky J, Te HS, Faust TW, Cohen SM. Varicella infection following varicella vaccination in a liver transplant recipient. Am J Transplant. 2002 ; 2 : 880-882
7. Chaves Tdo S, Lopes MH, de Souza VA, Dos Santos Sde S, Pereira LM, Reis AD, David-Neto E. Seroprevalence of antibodies against varicella-zoster virus and response to the varicella vaccine in pediatric renal transplant patients. Pediatr Transplant 2005 ; 9 : 192-196
8. Weinberg A, Horslen SP, Kaufman SS, Jesser R, Devoll-Zabrocki A, Fleckten BL, Kochanowicz S, Seipel KR, Levin MJ. Safety and immunogenicity of varicella-zoster virus vaccine in pediatric liver and intestine transplant recipients. Am J Transplant 2006 ;6 : 565-568
9. Khan S, Erlichman J, Rand EB. Live virus immunization after orthotopic liver transplantation. Pediatr Transplant 2006 ; 10 : 78-82
10. Kraft JN, Shaw JC. Varicella infection caused by Oka strain vaccine in a heart transplant recipient. Arch Dermatol 2006 ; 142 : 943-945
11. Shinjoh M, Miyairi I, Hoshino K, Takahashi T, Nakayama T. Effective and safe immunizations with live-attenuated vaccines for children after living donor liver transplantation. Vaccine 2008 ; 26 : 6859-6863
