# Supplementary material for: Prospective study of live attenuated vaccines for patients receiving immunosuppressive agents
Source: PLoS One. 2020 Oct 1;15(10):e0240217. doi: 10.1371/journal.pone.0240217 (PMC7529194; doi:10.1371/journal.pone.0240217)
Supplement: S2 File — (DOCX) [file pone.0240217.s002.docx]

**研究課題名：免疫抑制薬内服中の患者への弱毒生ワクチン接種についての前方視的研究**

**1. 研究要旨**

免疫抑制薬内服中は弱毒生ワクチンは接種禁忌となっている。しかしながら、免疫抑制薬内服中の患者は、水痘や麻疹などのウイルス感染が重症化するリスクが高く、可能であれば生ワクチンを接種して免疫を獲得しておくのが望ましい。本研究は、免疫抑制薬内服中で、麻疹、風疹、水痘、ムンプスのいずれかの抗体が（－）または（±）（EIA-IgG<4）事前の免疫検査（CD4細胞数、PHA lymphocyte stimulation test、血清IgGなど）を行い、条件満たしたことを確認して、弱毒生ワクチン（MRワクチン、水痘ワクチン、またはムンプスワクチン）を行い、2～6ヶ月後に当該ワクチンのウイルス抗体価および有害事象を評価する、単施設における前向き介入研究である。

**2. 研究責任者**

国立成育医療研究センター　腎臓・リウマチ・膠原病科　　亀井宏一

**3. 共同研究者**

国立成育医療研究センター　腎臓・リウマチ・膠原病科　　　小椋雅夫

国立成育医療研究センター　感染症科　　　　　　　　　　　　　宮入烈

国立成育医療研究センター　感染症科　　　　　　　　　　　　　庄司健介

国立成育医療研究センター　消化器科　　　　　　　　　　　　　新井勝大

国立成育医療研究センター　総合診療部　　　　　　　　　　　 伊藤玲子

北里大学　小児科　　　　　　　　　　　　　　　　　　　　　　　　　　石倉健司

横浜市立大学　小児科　　　　　　　　　　　　　　　　　　　　　　　伊藤秀一

**4. 研究の背景**

American Academy of PediatricsのRed Book^1)^では弱毒生ワクチン接種は免疫抑制薬中止後3ヶ月は避けるべきであると書かれている。また、我が国の免疫抑制薬（タクロリムス、シクロスポリン、ミゾリビン、アザチオプリン、ミコフェノール酸モフェチル、メトトレキサート）の添付文書にも、併用禁忌薬として弱毒生ワクチンが記載されている。

免疫抑制療法中あるいは免疫不全患者はウイルス感染症が重篤化するリスクが高い。特に、水痘は免疫抑制状態だと内臓臓器障害による多臓器不全を合併することがあり、当センターでも院内感染の水痘による多臓器不全で救命できなかった症例を経験している。米国と異なり麻疹や水痘が社会的に流行することが少なくない我が国では、これらのウイルス感染のリスクに常にさらされている。長期に免疫抑制薬の内服をせざるを得ない子供たちを、こうしたウイルス感染症から守るのは我々の責務であると考える。

一方、免疫抑制薬内服中の患者への弱毒生ワクチンの接種について、これまで数多くの報告がなされており、ケースシリーズについてまとめたものを資料２に示す^2)-11)^。ほとんどが臓器移植後で全例免疫抑制薬を内服している状況であるが、これらの報告を合計すると、計192接種で、146名が抗体を獲得しており、抗体獲得率は76％であった。また有害事象については、192接種中急性拒絶1名（0.5%）、ワクチン株によるウイルス感染発症8名（7名水痘、1名流行性耳下腺炎）（4.2%）、発熱5名（2.6%）、局所反応8名（4.2%）で、致命的な有害事象はなかった。このワクチン株によるウイルス感染の発症率（4.2%）は、健常人における水痘ワクチン後の水痘発疹の発症率（3.8%）と変わりはない。一般的に、ワクチン株でウイルス感染症が発症したとしても、通常の罹患に比べて軽症である。

難治性ネフローゼ症候群患者（ステロイド抵抗性ネフローゼ症候群あるいは頻回再発型ネフローゼ症候群）はネフローゼ症候群全体の約4割を占め、比較的低年齢発症が多く、乾燥弱毒生麻疹風疹混合ワクチンや乾燥弱毒生水痘ワクチンなどの生ワクチンが未接種の患者が少なくない。難治性ネフローゼ症候群患者はシクロスポリンやミゾリビンなどの免疫抑制薬を漸減・中止すると再発を繰り返すことが多く、免疫抑制薬の中止が極めて困難であるのが現状である。また、腎移植を受ける患者は全例生ワクチンを移植前に接種し、抗体の上昇を確認して腎移植を行っている。移植後は、終生免疫抑制療法を継続することとなる。免疫抑制療法を継続することで、その後抗体が消失することも少なくなく、その際はウイルス感染のリスクにさらされることとなる。従って、こうした免疫抑制薬を中止することが困難な子供たちをウイルス感染症から守るためには、病状が安定している時期に弱毒生ワクチンを接種することが必要不可欠である。

**5. 研究目的**

本研究の目的は、小児腎疾患、小児膠原病疾患、小児消化器疾患、腎移植を受けた患者などで免疫抑制薬を内服中で、一定のレベルの免疫機能が保たれている患者に、病状が安定している時期に抗体未獲得の弱毒生ワクチンを接種し、その効果と安全性を評価することである。

**6. 研究デザイン**

本研究は単施設における前向き介入研究である。

**7. 対象患者**

＜対象疾患＞

腎疾患患者（ネフローゼ症候群、慢性糸球体腎炎など）

腎移植を受けた患者

膠原病患者（全身性エリテマトーデス、若年性リウマチなど）

消化器疾患患者（炎症性腸疾患など）

肝疾患患者（自己免疫性肝炎など）

その他当センターで診療を受けている免疫抑制薬内服中の患者

＜接種条件＞

1. 1歳以上である。
2. タクロリムス、シクロスポリン、ミゾリビン、アザチオプリン、ミコフェノール酸モフェチル、メトトレキサート、エベロリムスのいずれかまたは複数の免疫抑制薬を内服中である。
3. 細胞性免疫マーカーが正常値である^38)^。

・CD4細胞数500/mm^3^以上

・PHAリンパ球幼若化反応のstimulation indexが101.6以上

1. 血清IgGが300 mg/dL以上である。
2. ステロイド投与が、プレドニゾロン1mg/kg連日未満あるいは2mg/kg隔日未満である。
3. タクロリムストラフ濃度が10ng/mL未満あるいはシクロスポリントラフ濃度が100ng/mL未満である。
4. 腎移植を受けた患者であれば、移植後1年以上経過しており、かつ急性拒絶反応を6ヶ月未満に起こしていない。
5. 原疾患の病勢が安定しており、接種によって原疾患が悪化するリスクが低いと判断されている。
6. 免疫抑制薬の中止が困難である。
7. 「免疫抑制薬内服患者への弱毒生ワクチン接種適応評価委員会」（資料7）で接種の医学的妥当性を検討し、接種の適応ありと判断されている。

**8. 研究方法**

患者または家族の同意を得た上で麻疹、風疹、水痘、流行性耳下腺炎の4種のウイルスの抗体価および下記に示す細胞性免疫能の評価を行う。抗体が未獲得（-または±、すなわちELISAのIgG 4.0未満）であるウイルスについて、接種することのメリット（抗体獲得）、デメリット（ワクチン株によるウイルス感染の発症や重篤な副作用の可能性など）、薬剤の添付文書では禁忌と書かれていることなどを十分に説明し、これらの内容を十分に理解されていることを確認し、インフォームドコンセントを書面で得て、さらに「免疫抑制薬内服患者への弱毒生ワクチン接種適応評価委員会」にて承認された上で、乾燥弱毒生麻疹風疹混合ワクチン、乾燥弱毒生麻疹ワクチン、乾燥弱毒生水痘ワクチン、乾燥弱毒生おたふくかぜワクチンのいずれかの弱毒生ワクチンの接種を行う。特に麻疹や水痘は免疫抑制療法中の患者が罹患すると致命的になるリスクが高いため、積極的に接種する。なお、ワクチン接種後、次のワクチン接種までは1ヶ月あけるものとする。接種後2-6ヶ月以上たった時点で、抗体獲得の有無を評価する。ELISA法によるIgGで4.0以上（＋以上）を抗体獲得と判断し、有効性ありと判定する。

有害事象についての評価として、24時間以内の即時型アレルギー反応の有無、ワクチン株によるウイルス感染の発症の有無などを評価する。すべての症例について、接種後の安全性についての評価を「免疫抑制薬内服患者への弱毒生ワクチン接種適応評価委員会」にて行う。なお、重篤な有害事象発症時は、同委員会を直ちに開催し、総長および倫理委員会に速やかに報告した上で、適切な対処を行うこととする。また、研究全体の中止の必要性について検討する。

＜免疫学的評価＞

１．液性免疫

・血清IgG値を接種前に提出（300mg/dLで接種可能）

・麻疹・風疹・水痘・流行性耳下腺炎の各種抗体価（ELISAによるIgG値）：接種前および接種2-6ヶ月後に提出

２．細胞性免疫

・CD4陽性細胞数を接種前に提出（500/mm^3^以上で接種可能）

・PHAによるリンパ球刺激試験を接種前に提出（Stimulation indexが101.6以上で接種可能）

**9. 評価項目**

1. 主要評価項目
2. 各種ワクチン毎の抗体獲得率の評価
3. 各種ワクチン毎の有害事象の頻度
4. 副次評価項目
5. Vaccine failure例の原因解析
6. 有害事象発症例の原因解析
7. 各ウイルス感染に対する予防効果

**10. 本研究の予測される成果**

当研究の有効性および安全性が認められれば、免疫抑制薬内服中の患者に対する生ワクチンの接種が可能となり、ウイルス感染の脅威から子供たちを守ることが可能となる。

**11. 研究期間**

2012年5月1日～2018年4月30日

**12. 研究倫理**

1. 研究参加の自由と撤回権

研究参加は、患者の自由意思で決定する。研究の説明は担当医が行い、同意を取得する。この際、説明書・同意書を添付する。患者が撤回を希望された場合はいつでも撤回が可能である。また、データが集計される前であれば、データの削除も可能である。検体についても同様である。研究に参加されない場合でも、診療上不利益を被ることはない。

1. 研究参加のメリット・デメリットについてのインフォームドコンセント

研究の説明を行う際、担当医は下記のメリット・デメリットを説明する。個々の症例を十分に吟味し、メリットがデメリットを上回ると判断された症例で、かつ御家族が十分にデメリットを理解された場合のみ、ワクチンの接種を行うこととする。

＜メリット＞

個々のウイルスに対する免疫状態の評価ができる。

ワクチンを接種することでウイルスの免疫を獲得し、感染のリスクを減らすことができる。

＜デメリット＞

- 1. 接種によりウイルス感染症を発症する可能性がある。
  2. 非常に重篤な副作用の可能性がある。
  3. 疾患によっては原疾患が悪化する可能性がある（例：ネフローゼ症候群の再発、移植腎の拒絶反応など）。
  4. 免疫抑制薬内服中であるため抗体の獲得率が低い可能性がある。

**13. 費用負担および賠償・補償責任について**

研究参加者は、ワクチン接種の費用は負担していただくことになる。また、ワクチン接種前の免疫の検査やワクチン接種の効果をみるための検査についての費用は通常の保険診療内で行われる。何らかの健康被害が生じた場合には、病院は必要な診察と治療を適切に行う。なお、重篤な有害事象発生時の補償として、研究期間中は株式会社損害保険ジャパンの臨床研究賠償責任保険（補償金額：死亡700万円～1億円、後遺障害1600万円～3000万円）に加入し、その保険料は「A grant from the National Center for Child Health and Development [grant number 24-10]」で負担するものとする。

**14. 個人情報の保護・研究成果の取扱い**

プライバシーの保護には十分配慮をし、成果を公表する場合には患者を同定できるような情報を一切含めず、匿名化を行い個人情報保護を行う。また、患者の検体は、患者識別対応表を作ることによって匿名化され、その対応表は当院の個人情報管理者によって管理され、他の人がアクセス出来ないようにする。なお、研究成果の報告の際は匿名化された結果のみを用いることで個人情報が漏出しないようにする。

**15. 引用文献**

1. Red Book. 2009 report of the Committee on Infectious Disease. American Academy of Pediatrics.
2. Danerseau AM, Robinson JL. Efficacy and safety of measles, mumps, rubella and varicella live viral vaccines in transplant recipients receiving immunosuppressive drugs. World J Pediatr 2008 ; 4 : 254-258
3. Rand EB, McCarthy CA, Whitington PF. Measles vaccination after orthotopic liver transplantation. J Pediatr 1993 ; 123 : 87-89
4. Zamora I, Simon JM, Da Silva ME, Piqueras AI. Attenuated varicella virus vaccine in children with renal transplants. Pediatr Nephrol 1994 ; 8 : 190-192
5. Kano H, Mizuta K, Sakakihara Y, Kato H, Miki Y, Shibuya N, Saito M, Narita M, Kawarasaki H, Igarashi T, Hashizume K, Iwata T. Efficacy and safety of immunization for pre- and post- liver transplant children. Transplantation 2002 ; 74 : 543-550
6. Levitsky J, Te HS, Faust TW, Cohen SM. Varicella infection following varicella vaccination in a liver transplant recipient. Am J Transplant. 2002 ; 2 : 880-882
7. Chaves Tdo S, Lopes MH, de Souza VA, Dos Santos Sde S, Pereira LM, Reis AD, David-Neto E. Seroprevalence of antibodies against varicella-zoster virus and response to the varicella vaccine in pediatric renal transplant patients. Pediatr Transplant 2005 ; 9 : 192-196
8. Weinberg A, Horslen SP, Kaufman SS, Jesser R, Devoll-Zabrocki A, Fleckten BL, Kochanowicz S, Seipel KR, Levin MJ. Safety and immunogenicity of varicella-zoster virus vaccine in pediatric liver and intestine transplant recipients. Am J Transplant 2006 ;6 : 565-568
9. Khan S, Erlichman J, Rand EB. Live virus immunization after orthotopic liver transplantation. Pediatr Transplant 2006 ; 10 : 78-82
10. Kraft JN, Shaw JC. Varicella infection caused by Oka strain vaccine in a heart transplant recipient. Arch Dermatol 2006 ; 142 : 943-945
11. Shinjoh M, Miyairi I, Hoshino K, Takahashi T, Nakayama T. Effective and safe immunizations with live-attenuated vaccines for children after living donor liver transplantation. Vaccine 2008 ; 26 : 6859-6863
